# Molecular Genetics and Genetic Testing in Myotonic Dystrophy Type 1

**DOI:** 10.1155/2013/391821

**Published:** 2013-03-18

**Authors:** Dušanka Savić Pavićević, Jelena Miladinović, Miloš Brkušanin, Saša Šviković, Svetlana Djurica, Goran Brajušković, Stanka Romac

**Affiliations:** Center for Human Molecular Genetics, Faculty of Biology, University of Belgrade, Studentski trg 16, P.O. Box 52, 11000 Belgrade, Serbia

## Abstract

Myotonic dystrophy type 1 (DM1) is the most common adult onset muscular dystrophy, presenting as a multisystemic disorder with extremely variable clinical manifestation, from asymptomatic adults to severely affected neonates. A striking anticipation and parental-gender effect upon transmission are distinguishing genetic features in DM1 pedigrees. It is an autosomal dominant hereditary disease associated with an unstable expansion of CTG repeats in the 3′-UTR of the *DMPK* gene, with the number of repeats ranging from 50 to several thousand. The number of CTG repeats broadly correlates with both the age-at-onset and overall severity of the disease. Expanded DM1 alleles are characterized by a remarkable expansion-biased and gender-specific germline instability, and tissue-specific, expansion-biased, age-dependent, and individual-specific somatic instability. Mutational dynamics in male and female germline account for observed anticipation and parental-gender effect in DM1 pedigrees, while mutational dynamics in somatic tissues contribute toward the tissue-specificity and progressive nature of the disease. Genetic test is routinely used in diagnostic procedure for DM1 for symptomatic, asymptomatic, and prenatal testing, accompanied with appropriate genetic counseling and, as recommended, without predictive information about the disease course. We review molecular genetics of DM1 with focus on those issues important for genetic testing and counseling.

## 1. Introduction

Myotonic dystrophy type 1 (DM1, MIM 160900) is the most frequent adult-onset muscular dystrophy. It was first clinically recognized by Steinert [1] and Batten and Gibb [2] in 1909. The main characteristics of DM1 are myotonia, progressive muscle weakness and wasting, and a broad spectrum of systemic symptoms [3]. Its clinical expression is unusual, characterized by a marked variability between and within pedigrees [3, 4] and a striking genetic anticipation [5] where the age-at-onset typically decreases by 25 to 35 years per generation [6]. Based on clinical ascertainment, worldwide prevalence is estimated to be 12.5/100000 [3], but it can be higher as many patients in older generation remain undiagnosed. 

DM1 is inherited in an autosomal dominant pattern. and the underlying mutation is an unstable expansion of CTG repeats in the 3′ untranslated region (3′UTR) of the dystrophia myotonica protein kinase gene (*DMPK*, MIM* 605377) [7–9] and in the promoter of the downstream SIX homeobox 5 gene (*SIX5*, MIM* 600963) [10]. Based on the nature of the causing mutation, DM1 belongs to “disorders of unstable repeat expansion” [11, 12]. Being the first disease described with an RNA gain-of-function mutation effect [13], DM1 is now the paradigm for RNA toxicity model of the disease pathogenesis, as reviewed elsewhere [14–16].

We review the molecular genetics of DM1 with the focus on the unstable nature of the underlying mutation in germline and soma, its relationship to clinical presentation of the disease, and implications for genetic testing and counseling.

## 2. Clinical Characteristics of DM1

DM1 is one of the most variable inherited human disorders, as the phenotypic expression varies from asymptomatic adults to severely affected neonates with congenital onset of the disease [3]. According to the age-at-onset and severity of symptoms, the main clinical forms of DM1 are late-onset/mild, adult-onset/classical, childhood-onset/juvenile, and congenital [3, 17]. Although each form presents specific clinical features, there is not absolute distinction between them, and they rather form a continuum.

Adult-onset form is the most prevalent with common age-at-onset in the second or third decade of life and clinical presentation typical for DM1 [3, 17]. Muscular symptoms include progressive muscle degeneration leading to weakness and wasting of facial, neck, and distal limb muscles, and extending more proximally in later stages. Myotonia, clinically presented as slowing relaxation of a normal muscle contraction, typically affects distal parts of the limbs as well as cranial and axial muscles. The most common systemic complications are cataract, cardiac conduction defects and arrhythmias, endocrine dysfunctions, gastrointestinal and respiratory involvement, frontal balding, axonal peripheral neuropathy, neurobehavioral manifestations, and cognitive impairment [3, 18]. Life expectancy is greatly reduced in adult-onset patients, particularly those with an early onset of the symptoms, and the most frequent causes of death are pneumonia and cardiac arrhythmias [19–21].

Congenital form is the most severe, often presenting *in utero *as polyhydramnios and with reduced fetal movement [22]. After delivery, the main features are severe generalized muscle weakness, hypotonia, and respiratory compromise. This severe muscle weakness is not caused by degenerative changes, as in the adult-onset form, but by developmental defects. Mortality in congenital form is usually caused by respiratory complications within the first year of life, with long-term ventilation having a poor prognosis [23]. Children without complicated neonatal course survive and have developmental delay [22]. Their hypotonia improves, they overcome motor delay and start to walk, but their intellectual development is poor and a majority of them are mentally retarded. Clinical myotonia and muscle degeneration usually appear late in childhood. Adults with congenital form have reduced survival by 50% beyond their mid-thirties [24]. The biphasic presenting of congenital form and etiology of the initial hypotonia with its associated problems are still poorly understood.

Childhood-onset form presents between one and ten years of age and is more clearly associated with cognitive and behavioral abnormalities, such as difficulties in learning and socialization at school [25]. In adolescence, myotonia is frequently present and distal muscular weakness may develop. In the second decade of life, individuals with childhood-onset form show many of the symptoms seen in the adult-onset form.

Late-onset form presents after the fifth decade of life with only mild symptoms, and individuals are often not aware of them [26]. Cataracts and baldness may be the sole symptoms. A late-onset muscle weakness may develop and myotonia may only be detectable by electromyography.

## 3. Genetic Studies in DM1 Pedigrees

Since 1918, it has been known that DM1 is inherited in an autosomal dominant pattern, but, interestingly, with an increased expressivity presenting as decreased age-at-onset and increased severity in subsequent generations of DM1 pedigrees, a phenomenon known as genetic anticipation [5]. This was in contrast to the fundamental principle of Mendelian genetics that mutation was stably transmitted between generations. The frequent observation in DM1 pedigrees is that grandparental generation shows cataracts with minimal or no neuromuscular involvement, the parental generation has more severe neuromuscular symptoms, while an affected child has early or congenital onset and is severely disabled [27]. Beside this, the influence of gender of the transmitting parent on disease course in child is also noticed. Children with the severe congenital form are born almost exclusively to affected mothers, at higher risk being women with neuromuscular involvement [28, 29]. An excess of mildly affected or asymptomatic fathers was found to transmit the disease in a clinically recognizable form [30–32]. When both parents of a DM1 patient are clinically normal, the odds are approximately 2 : 1 that the father is the transmitting parent [32].

## 4. Molecular Genetics of DM1

Understanding the puzzling genetic features in DM1 pedigrees and an extreme clinical variation of the disease became possible when the underlying mutation was revealed [7–9]. It turned out to belong, at that time, to a newly discovered type of mutation, referred to as dynamic mutation. Dynamic mutations are unstable changes (mostly increases/expansions) in the copy number of simple DNA repeats [33]. They are associated with at least 22 hereditary neurological diseases (e.g., Huntington disease, fragile X syndrome, spinal and bulbar muscular atrophy), known as “disorders of unstable repeat expansion”, as reviewed elsewhere [11, 12, 34]. 

Simple DNA repeats vary in copy number in normal individuals and are stably transmitted with an average mutation rate of ~10^−3^ per locus per gamete per generation [35]. For the loci undergoing dynamic mutations, expansions begin when the length of simple DNA repeats exceeds a threshold of ~100–150 bases. After this threshold is overcome, further expansions become progressively more likely to occur, leading to the accumulation of dozens to thousands of repeats in just a few generations [33, 36]. The mutation rate is related to an initial repeat copy number and can even reach the value of 1 per locus per gamete per generation, meaning that the repeat copy number is changed upon every intergenerational transmission. 

A considerable number of “disorders of unstable repeat expansion” are characterized by a specific genotype-phenotype correlation, such that the longer repeat arrays are associated with an earlier age-at-onset and with more severe symptoms [37]. The progressive nature of expansion process across generations, together with the characteristic genotype-phenotype correlation, provides a biological basis for a long-debated phenomenon of genetic anticipation, seen in a majority of the “disorders of unstable repeat expansion” [33, 37]. 

CTG repeat copy number in the *DMPK* gene is polymorphic in a general population, ranging from 5 to 35, and undergoes a pronounced expansion in DM1 individuals, ranging from 50 to several thousand [38]. The size of the unstable CTG repeats is roughly correlated with both the age-at-onset and overall severity of the disease [7, 39, 40]. Commonly, asymptomatic or late-onset DM1 individuals have from ~50 to 80 CTG repeats and these relatively small expansions are termed protomutations [41]. The upper limit of expansion size in the late-onset DM1 individuals is ~150 CTG repeats [38, 40]. Adult-onset DM1 individuals have a broad range of CTG repeat number, roughly between 100 and 1000 (mean size ~650 repeats), while congenital and childhood-onset DM1 individuals have more than 1000 repeats (mean size ~1200 repeats), [38, 40]. The expansions of more than 80 CTG repeats are known as disease-associated (full) mutations. *DMPK* alleles, which are between the normal and protomutation range (from 35 up to ~50 repeats) are very rare. They are usually not associated with the disease and are termed premutations [42]. 

Until recently the CTG array in the *DMPK* gene was assumed to be a pure tract (without interruptions/variant repeats), in contrast to a majority of other simple DNA repeats associated with “disorders of unstable repeat expansion.” However, 4-5% DM1 individuals carry interrupted expanded alleles with interruptions being multiple CCG triplets, CCGCTG hexamers or CTC triplets, all located at the 3′ end of the CTG array [43, 44]. Variant repeats seem not to be to present in normal DM1 alleles.

### 4.1. Intergenerational Change in Repeat Copy Number and Parental-Gender Effect in DM1

In DM1 pedigrees intergenerational change in repeat copy number is biased toward further expansion [32, 39, 40, 45, 46], but less frequently contraction [47, 48], and extremely rare reversion can occur [49]. The direction and extent of intergenerational change in repeat copy number depend on both parental expansion size and gender of the transmitting parent. There is a wide correlation between the size of an expanded allele in parent and the change in the expansion size when it is transmitted to the offspring. 

Premutation and protomutation are inherited stably or with smaller changes in repeat copy number for several generations if transmitted by female. When transmitted by men, premutation shows increased instability toward expansion, even reaching the full mutation in a single generation, while protomutation almost invariably results in a large increase in repeat copy number [32, 41, 50–52]. For example, average intergenerational expansion in DM1 alleles with less than 100 repeats was 310 repeats in male transmission versus 105 repeats in female transmission, and the expansions with more than 100 repeats occurred in 92% in paternal transmissions compared to 44% in maternal transmissions [32]. A marked expansion-biased instability of premutation and protomutation upon male transmission is the molecular basis for an excess of males in the last asymptomatic generation in DM1 pedigrees [30–32].

Disease-associated DM1 alleles are almost always unstably transmitted by both genders. For alleles with repeat copy number ranging from 200 to 600 the most frequent event is further increased in repeat copy number [39, 40, 45], but contraction [47, 48] and extremely rare reversion can occur [49]. The absolute increase in repeat copy number is greater in female transmission (mean size ~500–600 repeats) than in male transmission (mean size ~260–280 repeats) [39, 40]. This difference may be the result of an expanded allele size in parent, which is, on average, larger in mothers than in fathers, and could be due to a sampling bias, since the families with the more severe forms, often inherited from the mother, are more likely to be recruited [40]. When the intergenerational increase is expressed as a proportion of the expansion size in parent, the gender difference is not longer seen [39]. Still, a study on the largest cohort of DM1 individuals (~1500) with the expansion size in the range from 200 to 800 repeats, showed that 66% of maternal transmissions resulted in expansions, whereas the majority (69%) of paternal transmissions resulted in stable transmissions or contractions [53]. 

The largest expansions, associated with congenital form of disease, are almost exclusively maternally transmitted [40, 45], though a few cases of paternally transmitted congenital form have been reported [54, 55]. Mothers of congenital offspring have an expanded allele size significantly greater than mothers of noncongenital offspring (mean size ~600 repeats versus ~250 repeats, resp.) [39, 40, 45]. However, less than 10% of affected mothers give birth to congenitally affected children [56]. In almost all cases affected sisters have children affected with the same DM1 form (either adult-onset or congenital), and the affected sibships, although with variable expansion size, present the same form of the disease [40]. These indicate the existence of still unknown familiar (genetic) factors which increase the risk of having a congenitally affected child. The extremely rare paternal transmission of congenital form can be associated with the observation that fathers with ~650 repeats occasionally pass a larger expansion to their offspring [39].

The estimated frequency of repeat contractions in DM1-pedigrees is 4.2–6.4% [48, 53]. They are usually associated with the change toward less severe or even asymptomatic DM1 form, but in some parent-child pairs anticipation is still present [48, 57]. Contractions are more frequently transmitted by males, and the occurrence of contraction in a family member increases the probability of another contraction in that DM1 pedigree. 

For alleles with similar expansion size, the parental-gender effect is comparable across different “disorders of unstable repeat expansion.” For example, expansions from premutation to full mutation in Huntington disease (HD) and a large expansions (up to ~100 repeat copy number) associated with a severe juvenile HD occur primarily upon male transmission [58–60], similarly to male-biased instability of DM1 premutations and protomutations. The large expansions in noncoding regions, associated with fragile X syndrome [61] and spinocerebellar ataxia type 8 [62], as well as with congenital form of DM1, are transmitted by females. 

Intergenerational changes in repeat copy number are a cumulative result of a pronounced germline and somatic instability of the CTG array in *DMPK* gene [57, 63, 64].

### 4.2. Germline Instability of Expanded DM1 Alleles

Germline and somatic instability of DM1 alleles was studied by small-pool PCR (SP-PCR), a method for detailed quantification of the degree of repeat size variation in a given sample [65]. SP-PCR analysis of sperm samples from DM1 individuals, who had either the adult-onset form or were asymptomatic, revealed a high level of repeat size heterogeneity in an individual, with mutation rate of almost 1 per gamete and a pronounced expansion bias [57, 66]. Allele size heterogeneity in sperm showed normal two-tailed distribution with a lower tail extending back down in the normal size range [57]. The degree of variation was highly variable between examined individuals and not obviously correlated with the progenitor allele size-one originally inherited by zygote and measured as the lower boundary of allele size distribution in blood [57, 66]. Two asymptomatic DM1 individuals with relatively stable protomutations in blood (75 and 90 repeats) showed the highest degree of repeat size heterogeneity in sperm (even exceeding 1000 repeats) and the most dramatic allele size increase (on average 180 repeats in one individual and, even, 700 in another). Four individuals with relatively small expansions in blood (average size from 200 to 300 repeats), and progenitor allele size from 150 to 200 repeats, had an average increase from 30 to 120 repeats in sperm. Two other individuals with similar progenitor allele size (210 and 390 repeats), but a higher average size in blood (630 and 700) showed an average increase of about 250 repeats in sperm. Finally, one individual with a highly variable blood distribution (ranging from ~190 to 700 repeats) showed a relatively low level of variation in sperm with the average size of 150 repeats. In all analyzed sperm samples alleles with more than 700–800 repeats were very rare [57, 63]. 

Among analyzed spermatozoa, left as unused preimplantation diagnostic material and taken from 10 DM1 individuals with progenitor allele size ranging from 62 to 256 repeats, 67% showed expansions with an average change of ~60 repeats, 14% showed contractions with an average change of ~40 repeats, whereas the repeats remained stable in ~10% of spermatozoa [67]. 

Studies on post-preimplatation diagnostic material, the unique opportunity to study instability in female gametes, revealed a significant increase in repeat length in immature and mature oocytes, which was about 10 times greater than in spermatozoa from DM1 individuals with similar allele size: 22 DM1 individuals with progenitor allele size ranging from 42 to 428 repeats showed an average increase of ~300 repeats in oocytes, while the minimal increase was ~100 repeats and the maximum one was 950 repeats [67]. In contrast to spermatozoa, contractions were not observed in oocytes.

Timing of germline instability of loci associated with “disorders of unstable repeat expansion” is not completely understood. In DM1 human oocyte increase in the repeat copy number was observed before completion of meiosis, either during premeiotic proliferation of oogonia or during prophase I [67]. In spinocerebellar ataxia type 1 (SCA1) transgenic mice, instability of the repeat copy number occurred while the oocytes were arrested in meiosis I after meiotic DNA replication [68]. In DM1 transgenic mice carrying more than 300 repeats in a large human genomic segment [69], expansions were present in spermatogonia and spermatozoa, indicating that they occurred at the beginning of spermatogenesis and that meiosis and postmeiotic mechanisms are probably not involved in germline expansions [70]. Similarly, expansions in the HD locus were observed in both premeiotic and postmeiotic human male germline cells [71], but in contrast experiments on HD transgenic mice revealed that expansion was a postmeiotic event occurring late in elongation of spermatids, as they become mature sperm cells [72]. 

Molecular mechanism of the germline instability of expanded alleles and, in general, of dynamic mutations are not well understood. The tendency of the repeat tract to expand or contract seems to be a function of its primary sequence, which enables formation of secondary hairpin-like structure [73, 74]. This intermediate can be formed during processes that involve the separation of DNA strands: DNA replication, repair, and recombination, and each of them has been implicated in repeat instability, as reviewed elsewhere [36, 75]. Several models support instability of expanded repeats during DNA replication [76, 77], while others suggest the appearance of errors in the DNA repair—mismatch repair system [78–80] or gap repair [72]. 

Different dynamics of DM1 instability in male and female germline is not influenced only by the absolute repeat size, but may also be governed by gender-specific factors that are included in DNA repair and/or replication and are important for mutational pathway. It was hypothesized that some kind of a selection operated to preclude expansions with more than 1000 repeats in spermatogenesis [40, 63], but it is still unclear why DM1 premutation and protomutation stay stable upon passage through female meioses, while full mutation in the same background undergoes dramatic expansion. 

### 4.3. Somatic Instability of Expanded DM1 Alleles

DM1 repeat size heterogeneity in somatic tissues (somatic mosaicism) is tissue specific [63, 81, 82], biased toward further expansions and continuous throughout the life of an individual [57, 64, 83]. 

Somatic mosaicism has been observed among a number of different tissues, and regarding those relevant to DM1 pathology, much larger expansions (2- to 13-fold greater) were found in skeletal muscles [57, 81, 82, 84] and heart [63] than in peripheral blood, while the smallest expansions were present in frontal cortex and thalamus [63]. In DM1 human fetuses the largest expansions occurred in heart, skin, skeletal muscle, brain, and kidney, and the smallest one in blood [40, 63, 82, 85, 86]. Since the repeat size heterogeneity was observed between tissues from 16-week-old fetus [86] and no mosaicism was detected in any of the affected embryos obtained as post-preimplantation diagnostic material [67], it is thought that somatic mosaicism in affected fetuses starts during the second trimester of gestation [85]. As proposed by Wöhrle et al. [85], this timing correlates with the period of onset of rapid growth of the fetus and implies that during differentiation period in the first trimester, the number of repeats is somehow stabilized. As differences between tissues do not appear to reflect the number of cell divisions during development, the timing of somatic mosaicism in human fetuses might be due to a greater fidelity of DNA repair mechanisms during differentiation period, which could not be sustained during the rapid growth phase and/or by the suppression of repeat expansion through methylation of the DM1 repeat region [85]. 

Somatic mosaicism of DM1-expanded alleles within one tissue is seen as a diffused band or smear on Southern blot analysis, or as discrete bands heterogeneous in size on SP-PCR analysis [57, 64]. Quantitative measurement of the repeat size heterogeneity in blood samples by SP-PCR showed a high level of variation, with the mutation frequency from 50% to over 90%, and an allele size distribution skewed towards larger alleles with a lower boundary, below which variant alleles are rare [57]. Data from population-based mathematical modeling of DM1 expanded alleles in blood suggest that bias towards the expansion is a cumulative effect of many expansion and contraction events, possibly as frequently as every other day [87]. The distribution skewed toward expansions is in agreement with the increase of the allele size heterogeneity and the mean allele size with the age of patient observed in the longitudinal studies [83], and together they indicate that somatic instability is continuous throughout life of an individual and expansion-biased. Different DM1 allele size distribution between blood and male germline implicates the distinct mutational dynamics in these tissues. 

The major factors affecting the level of somatic instability throughout life are the progenitor allele size and age-at-sampling [83], which together account for 89% of the observed variation [88]. Residual variation is individual specific and heritable as quantitative trait, which implicates the role of transacting factors as modifiers of somatic instability [88]. The most obvious candidates for transacting modifiers are components of the DNA mismatch repair pathway, since mismatch repair genes have been shown to be critical for generating somatic CTG-CAG repeat expansions in mice [79, 89, 90]. Estimation that many expansion and contraction events could occur as frequently as every other day additionally supports the role of DNA repair or transcription, rather than DNA replication, in somatic instability [87].

### 4.4. Genotype-Phenotype Correlation in DM1

Significant correlations have been reported between genotype and age-at-onset of DM1 [39, 53, 91, 92]. Correlations with specific DM1 symptoms (e.g., neuromuscular involvement [93, 94], cardiac involvement [95–97], cognitive impairment [98, 99], mortality [100], and peripheral neuropathy [101]) are often poor or absent. However, the largest examined cohort of DM1 patients (2650 individuals) revealed the correlation between expansion size and overall DM1 clinical phenotype: (i) patients with an expansion up to 100 repeats have almost 100% probability to stay asymptomatic with normal or abnormal EMG (F0 phenotype) or to develop minimal signs of myotonia with EMG abnormalities (F1 phenotype); (ii) patients with 100–350 repeats have a ~95% probability to develop F1 phenotype or more severe F2 phenotype characterized by myotonia and distal weakness, ECG abnormalities, gonadal dysfunction, mild mental retardation, glucose intolerance, and cataract; (iii) patients with 450–1800 repeats have a 85–95% probability of developing F2 phenotype or the most severe F3 phenotype marked by proximal weakness, cardiac involvement, endocrine dysfunction, mental retardation and cataract; (iv) patients with more than 2000 have a ~90% probability of developing F3 phenotype [53]. 

Nevertheless, genotype-phenotype correlations in DM1 are compromised due to potential inaccuracy in phenotypic data [17] and by tissue-specific, expansion-biased somatic instability of mutant alleles over the life of a patient [57, 64, 83]. Namely, larger mutant alleles are present in the primarily affected skeletal muscle tissue rather than in blood [81, 84], and there is a difficulty in eliminating the effect of the patient age-at-sampling. Additionally, somatic expansions are assumed to contribute to tissue-specificity and progressive nature of the symptoms [83, 88, 102, 103]. Also, there are technical difficulties for precise assessment of the number of CTG repeats using Southern blot hybridization of genomic DNA, as well as a disagreement in published data as to which point of the diffuse smear on the blot (representing alleles of a different size due to somatic instability) is the appropriate allele size to be used in genotype-phenotype correlation [38–40, 94]. This can be overcome by applying SP-PCR analysis and using the lower boundary in the allele size distribution in blood as progenitor allele. This is a good estimate for the progenitor allele size, since the lower boundary is conserved between tissues and specific for a DM1 individual, and blood is apparently one of the most stable tissues [57, 66]. Even with this approach in some older individuals with larger alleles, it is possible that DM1 alleles in all cells may have expanded beyond the progenitor allele length [83], and it is likely that, in some younger individuals, the lower boundary of the distribution may drop below the progenitor allele due to contractions [87].

By defining DM1 expansion in blood by three parameters: progenitor, average, and largest allele size and by using the SP-PCR, there was reported a negative linear correlation of age-at-onset and average expansion size in juvenile-adult DM1 individuals whose progenitor allele is less than 245 repeats long [103]. This result favors the hypothesis about the existence of a threshold beyond which an increase in repeat length makes no additional contribution toward age-at-onset [91]. However, recent study on a large cohort of DM1 individuals showed that the estimated progenitor allele length was the major modifier of age-at-onset of the disease, accounting for 64% of the variation, without the threshold above which repeat length did not contribute toward age-at-onset [88]. Age-at-onset is further modified by the level of individual-specific somatic instability: patients in whom the number of repeats expands more rapidly have an earlier age-at-onset [88]. Somatic instability of expanded alleles over life has also been implied in the progression of neuromuscular symptoms in juvenile-adult DM1 individuals [103].

Somatic instability has also compromised attempts to precisely measure intergenerational repeat dynamics. Namely, intergenerational change of the repeat length determined by measuring the blood allele size in parent and offspring usually correlates quite well with the observed anticipation. However, a relatively high proportion of cases with apparent contraction in the repeat length still show the anticipation [48, 66]. In these cases progressive age-depended somatic instability in blood masks the true germline expansion, and such intergenerational change is termed “pseudocontraction” [57]. 

### 4.5. Effects of the Interruptions on Repeat Stability and Phenotypic Manifestation in DM1

Interruptions (CCG, CTGCCG, or CTC repeats or even nonrepetitive DNA sequences) at the 3′ end of the CTG array in DM1 alleles with more than 35 repeats may have considerable consequences on mutational dynamics and may also affect phenotypic manifestations [43, 44, 104]. Upon transmission, the interruptions in DM1-expanded alleles show instability and substantial intrafamilial variability, in both their number and location among the relatives [43, 44]. The exception was one pedigree where a complex interruption was stably transmitted [44]. The interruptions may have stabilizing effect on somatic and germline instability, as relatively small maternal intergenerational expansions were observed, or could even predispose to germline contractions, since the frequency of the intergenerational contractions was higher than expected for DM1 and transmitted by females [43, 44]. This can explain the absence of congenital form in the examined families with interrupted DM1 alleles where the disease was maternally transmitted. 

DM1-expanded alleles with interruptions may be associated with an atypical phenotype, though the family with cosegregating DM1, Charcot-Marie-Tooth neuropathy, encephalopathic attacks and an early hearing loss is the unique example [44]. In families studied by Musova et al. [43] phenotype of the patients did not differ significantly from the typical clinical picture of DM1. However, in some cases muscular dystrophy was absent and the later age-at-onset than expected solely from the expanded allele size was seen. Interestingly, two of the patients also presented with a polyneuropathy.

Intriguingly, the interruptions were observed in the extremely rare premutations, even in four males. The individuals with interrupted premutations should be unaffected based on their repeat length alone, but this was the case only in one male with 37 interrupted repeats [105]. Other two males, with 37 and 43 interrupted repeats, had a neuromuscular phenotype [43]. Although premutations tend to expand upon male transmission [50], allele with 43 interrupted repeats was stably transmitted. Analysis of a larger set of individuals is warranted to access the frequency of interrupted DM1 alleles and to determine their possible causal or modifying effect on DM1 phenotype. 

Identification of a tissue-specific CTG-free configuration in expanded allele in one juvenile-adult DM1 individual further broadens the possible unusual configuration of the expanded DM1 alleles [104]. Described insertion led to a complete loss of the CTG array, retaining only the first CTG and the TG of the very last CTG repeat in cerebral cortex, skeletal muscles, and cerebellum. However, the clinical significance of this distinct configuration in the DM1-expanded allele requires further analysis. 

## 5. Genetic Testing and Counseling in DM1

Identification of the causing DM1 mutation enabled an accurate and specific genetic test to be routinely used in diagnostic procedure. However, genetic counseling in DM1 is still very complex, due to a highly variable clinical presentation, in both in severity and age-at-onset, anticipation, and influence of gender of the transmitting parent.

### 5.1. Molecular Diagnostic Tests in DM1

**Figure 1:**
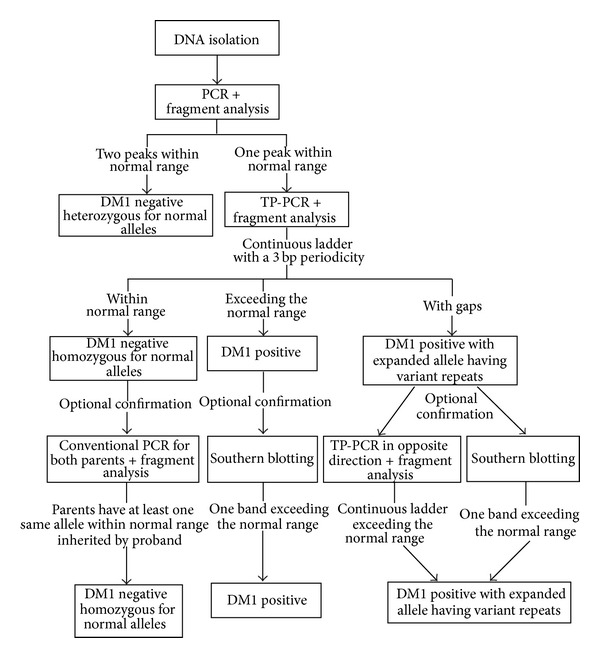
Flow diagram of a genetic test on myotonic dystrophy type 1 (DM1). A two-step procedure is used in DM1 genetic testing. The first step is PCR followed by fragment length analysis, which identifies and sizes alleles within normal range. The second step employs one of the techniques which differentiates between individuals who are homozygous for an allele within normal range and DM1 individuals carrying one allele within normal range and one unamplifiable expanded allele. The most widely used technique in the second step is the triplet-repeat primed PCR (TP-PCR), which utilizes locus-specific PCR primers in combination with a primer designed across the repeated sequence, and provides no size estimation, but rather a simple “present”/“absent” result for an expanded allele. After the fragment length analysis step, products of different sizes are visible as a continuous ladder with a 3-base-pair periodicity. In the presence of a DM1-expanded allele, a continuous ladder exceeds the normal size range. The lower part of the flow diagram shows optional methods used to confirm the obtained result of the two-step diagnostic procedure for DM1, employed when some samples show inconclusive findings. Applied together, PCR, TP-PCR, and Southern blotting methods provide high sensitivity and specificity, and diagnostic laboratories should have a facility to use more than only one methodological approach (usually TP-PCR and one of the Southern blot methods).

Two-step molecular diagnostic procedure is used in DM1 genetic testing (Figure 1) [106]. The first step is to analyze whether an individual is heterozygous for alleles within normal size range by using PCR and fragment-length analysis. If only one normal allele is detected, one of subsequent techniques are used to detect or exclude possible DM1 expansions. For many years Southern blotting of genomic DNA [107] or Southern blotting of long-range PCR products [108] has been used. Recently, triplet-repeat primed PCR (TP-PCR) [109] has come into routine diagnostic procedure [110]. 

Southern blotting of genomic DNA is time-consuming procedure and requires a considerable amount of high quality DNA. On the other hand, it gives information about repeat copy number and has no limitation to detect even the largest expansions. Southern blotting of long-range PCR products, optimized for amplification of a long and GC rich template, requires less DNA, as small as 15 pg [108], even of lower quality, and gives reliable information about repeat copy number with using a few replicate of PCRs with 180–300 pg of DNA [88]. However, it is also time-consuming and may fail to amplify the largest expansions. TP-PCR is a faster technique, whose specificity and sensitivity is almost 100%, even with the DNA isolated from a single cell, making it usable in preimplantation diagnostics [111]. It is based on the use of locus-specific PCR primers in combination with a primer designed across the repeated sequence [109]. After PCR and fragment analysis, products of different sizes are visible as continuous ladder with a 3-base-pair periodicity. In the presence of DM1 expansion, a continuous ladder exceeds the normal size range. This method provides no size estimation at all, but rather a simple “present”/“absent” result for an expanded allele. If used with a primer located downstream of the CTG repeat, it can be useful for detection of variant repeats located at the 3′ end of CTG array, when gaps could be observed in the regular and contiguous peak pattern, but the reaction may sometimes fail, leading to false negative results [43, 112]. TP-PCR in opposite direction or alternative Southern blotting methods can overcome this situation. This is the reason why performance of TP-PCR in both directions is suggested in order to increase its reliability and accuracy for DM1 testing. 

Used together, the aforementioned techniques provide high sensitivity and specificity. As some samples may show inconclusive findings with just one method, diagnostic laboratories should have facility to use more than one methodological approach (usually TP-PCR and one of the Southern blot methods).

### 5.2. Indications for DM1 Genetic Testing

Genetic testing can be confirmatory/symptomatic testing, preclinical/asymptomatic testing, prenatal testing, and preimplantation testing. The main indications for appropriate kind of genetic testing in DM1 were given by The International Myotonic Dystrophy Consortium (IDMC) [38] (Table 1). 

Genetic testing should be accompanied with appropriate genetic counseling. The result of symptomatic genetic testing has direct implications for other family members (siblings and children), and genetic counseling should be available to tested person and to any other interested family members. Individuals who have asymptomatic testing should always have genetic counseling by a qualified counselor, including pretest counseling to assure that the tested person understands the risks and benefits of testing. 

IDMC [38] recommendations for testing of minors are in agreement with many other policies regarding this issue [113]. Minors should not be tested unless there is a direct medical benefit, and this measure is to ensure that the tested person fully understands the risks and benefits of testing. Exceptions might be appropriate in the case of a symptomatic minor for whom confirmatory testing is necessary.

### 5.3. Reporting Guidelines

At the EMQN Best Practice Meeting, held in 2008 in Nijmegen (The Netherlands), a consensus for the optimal reporting guidelines in myotonic dystrophies was reached (Table 2), and subsequently published as “Best practice guidelines and recommendations on the molecular diagnosis of myotonic dystrophy types 1 and 2” [106]. 

The predictive clinical use of the genetic test result in DM1 is not recommended [38] and may be misleading for several reasons: (i) the distribution of the expanded DM1 alleles is widely spread out, and the expansion sizes are overlapped with each other in different DM1 clinical forms; (ii) genotype-phenotype correlation is compromised by the age-dependent, expansion-biased somatic mosaicism, which also influences the severity of the disease; (iii) apart from depending on the expanded allele size and age-at-sampling, somatic instability also depends on individual-specific factors. Therefore, it is not appropriate to offer a prognosis based on the expansion size after symptomatic testing nor to give information about the age-at-onset, the kind of symptoms, their severity, nor the rate of progression based on the repeat size after asymptomatic or prenatal testing. 

Estimating the risk of having congenitally affected offspring is complicated because of the fact that 18% of mothers with congenital offspring have a similar expansion size as mothers of noncongenital offspring (~less than 300 repeats). Moreover, there is the overlap in the range of expanded allele size between individuals with congenital and other DM1 forms [39, 40, 45]. However, the observations that affected sisters have children affected in almost all cases with the same DM1 clinical form [40] and that the affected sibships present with the same form of the disease could be useful in counseling.

### 5.4. Prenatal Diagnosis and Preimplantation Genetic Diagnosis in DM1

In families at risk to have a child with DM1, prenatal diagnosis (PND) can be offered. Prenatal samples are chorionic villi, taken between the 10th and 12th week of gestation, or amniotic fluid, taken between the 14th and 16th week of gestation. Analysis of DNA from the mother is also required to exclude maternal contamination in the fetal sample, especially if the chorionic villi sample is used. In some cases, usually when fetus is homozygous for DM1 alleles within normal range, analyses of DNA from the unaffected parent can be required to verify the PCR results. Indications for PND according to IDMC [38] are presented in Table 1.

Preimplantation genetic diagnosis (PGD) for DM1, an alternative to prenatal diagnosis for individuals at risk of transmitting DM1, was developed in 1995 [114] and is now offered routinely in several countries [115–117]. PGD involves the genetic testing of blastomeres from embryos obtained *in vitro*, followed by the transfer of only those diagnosed as healthy with regard to the disease under consideration [118]. So, unlike the PND which is followed by the termination of pregnancy in the case of an affected embryo, PGD circumvents the problem of therapeutic abortion. From the viewpoint of DNA analysis, prerequisite for a clinically applicable PGD for DM1 has been the development of a sensitive single-cell PCR assay. The first approach was based on the detection of embryos heterozygous for normal DM1 alleles, exploiting the feature that DM1 CTG repeats are highly polymorphic in a general population [38]. The originally applied DNA technique was a nested PCR [114] and was subsequently replaced by 1000 times more sensitive fluorescent PCR, which also reduced the rate of allelic drop out (failure to amplify one or two alleles in a heterozygous cell) from 21% to 5.2%, enabling a much smaller loss of embryos due to misdiagnosis [119]. As the expanded DM1 alleles were not amplified by aforementioned assays, the disadvantage of this approach was application only for informative couples—affected partner has a wild type allele clearly different in size from the unaffected one. The selected healthy embryos were always heterozygous, carrying the normal allele of the affected parent and one of the two normal alleles of the unaffected parent, while detecting only one DM1 allele from the unaffected parent (regardless of possible allelic drop out) indirectly meant that embryo was DM1 positive. From 1997 onwards, with the development of a sensitive TP-PCR for detection of DM1 expansions, PGD could also be offered for half-informative (couples with both partners sharing one normal allele of the same size) or noninformative couples (couples with three normal alleles identical in size) [111]. Further improvement of the accuracy of PGD for DM1, in terms of detection of contamination of the sample and allelic drop out, was achieved by the use of multiplex PCR with combined DM1-linked markers and detection of the repeat fragments [116]. 

Report on a large cohort of DM1 patients undergoing PGD for DM1-showed that (i) it was safe in DM1-affected women after careful pretreatment assessment with regard to disease-specific complications (cardiological, anaesthetical, and obstetrical problems), (ii) delivery rate per treatment cycle was 20%, with at least one baby after two PGD cycles in almost half of the couples, and (iii) the children born were generally in good health up to 2 years of age and comparable to children born after intracytoplasmatic sperm injection for infertility and after PGD for other genetic conditions [116]. As pointed out by de Rademaeker et al. [116], PGD for DM1 is a well-established procedure resulting in the birth of unaffected and mostly healthy children and should be considered as an alternative to PND in couples with concomitant infertility and couples unwilling to undergo termination of pregnancy (Table 1).

## 6. Conclusions

Discovering that an expansion of the CTG repeats in the DMPK gene is underlying DM1 mutation has opened molecular genetic studies and has facilitated the understanding of underlying pathogenic mechanisms of this disease. Many of its puzzling features, such as a striking genetic anticipation, parental-gender effect in DM1 pedigrees, tissue-specificity, and progressive nature of the disease have been explained by a characteristic mutational dynamics in male and female germline as well as in somatic tissues. Also, a highly variable phenotypic expression, varying from asymptomatic adults to severely affected children with congenital onset of the disease, is broadly correlated with the repeat copy number in mutated allele. However, diverse mutational dynamics of different kinds of DM1-expanded alleles in male and female germline are not completely understood. In addition, it is clear that the repeat copy number is not the only factor determining the phenotypic manifestation of the disease or the risk of having congenitally affected offspring. A new “next-generation” sequencing platform, single-molecule real-time (SMRT) sequencing, suited for sequencing of long, repetitive DNA sequence [120], is a promising approach for studying interruptions and epigenetic marks in the expanded DM1 alleles, as additional factors influencing germline and somatic repeat instability and phenotypic expression of the disease.

## Figures and Tables

**Table 1:** The main indications for genetic testing in DM1 given by The International Myotonic Dystrophy Consortium (IDMC) [38], complemented with the suggested indications for preimplantation genetic diagnosis [116].

Genetic testing	Indication for testing
Confirmatory or symptomatic	(i) To confirm the clinical diagnosis: the gene test will increase the physician's confidence in diagnosing a patient with typical symptoms.
	(ii) To clarify an uncertain/differential clinical diagnosis: the gene test will be useful for individuals in whom DM1 is part of a wider differential diagnosis.

Asymptomatic or preclinical	(i) To determine which progenitor has DM1 mutation, and this information is important in genetic counseling and carrier testing to the relevant side of the family.
	(ii) To modify *a priori* risk of inheriting the DM1 allele.
	(iii) To test asymptomatic parent who has 50% risk for DM1 and requires prenatal testing.*

Prenatal testing	(i) If a parent has already been diagnosed with DM1, genetic test can be used to assess fetal risk.
	(ii) If a parent is at 50% risk and asymptomatic, the best approach is a two-step process by which at-risk parent is tested first, and prenatal diagnosis is done subsequently (if still necessary).
	(iii) Prenatal diagnosis should not be considered if parents would have the child regardless the test result.

Preimplantation testing	(i) Alternative for prenatal testing.
	(ii) Couples with concomitant infertility.
	(iii) Couples unwilling to undergo termination of pregnancy.

*In addition to IDMC indications.

**Table 2:** Reporting guidelines for DM1 genetic testing according to Kamsteeg et al. [106] complemented with the influence of gender of the transmitting parent.

Genetic test result	Recommended reporting
No expansion-homozygous or heterozygous for allele in the size range of 5–35 repeats (normal alleles)	DM1 diagnosis is excluded; when it concerns a fetus, it is not affected.

	(i) DM1 diagnosis is excluded; when it concerns a fetus, it is not affected.
A heterozygous expansion in the size range of 36–50 repeats (premutation alleles)	(ii) Premutations may or may not expand in next generations. Transmission by female mostly results in stable inheritance or small changes in repeat copy number, while when transmitted by men, they are more prone to expand, even reaching the disease-associated mutation in a single generation, thus raising the risk of having affected child.
	(iii) Relatives (including offspring) of the counselee may be at risk of developing DM1 and should be offered counseling. An offer of repeat-length analysis to those relatives is warranted.

	(i) When symptoms are evident, the diagnosis of DM1 is confirmed.
	(ii) When symptoms of DM1 are not evident (asymptomatic family member or fetus), the individual is at risk of developing DM1, although individuals with a repeat expansion of this size may also remain symptomless.
A heterozygous expansion in the size range of 51–150 repeats	(iii) Counselees in the reproductive age is warranted. Smaller repeat expansion of this size range can be stably transmitted by female, while larger repeat expansion of this size range raising the risk of having a child with even congenital form of DM1. When transmitted by male repeat expansion of this size range almost invariably results in a large increase into the disease-associated mutation, raising the risk of having affected offspring.
	(iv) Relatives (including offspring) of the counselee may be at risk of developing DM1. Due to anticipation in DM1, offspring may be more severely affected. Relatives should therefore be offered counseling. An offer of repeat-length analysis to those relatives is warranted.

	(i) When symptoms are evident, the diagnosis of DM1 is confirmed.
	(ii) When symptoms of DM1 are not evident (asymptomatic family member), the individual is at risk of developing DM1, although individuals with a repeat expansion of this size range may rarely remain symptomless.
A heterozygous expansion with a size over 150 repeats	(iii) When it concerns a fetus, it is very likely to be affected and has a high risk to be more severely affected than the affected parent.
	(iv) Counselees in the reproductive age is warranted. Women are, especially, at risk of having children with the congenital form of DM1.
	(v) Relatives (including offspring) of the counselee may be at risk of developing DM1. Due to anticipation in DM1, the offspring may be more severely affected. Therefore, relatives should be offered counseling. An offer of repeat-length analysis to those relatives is warranted.
